# Readability Assessment of HPV Vaccination and Cervical Cancer Information: A Systematic Scoping Review

**DOI:** 10.3390/healthcare9101246

**Published:** 2021-09-22

**Authors:** Tsuyoshi Okuhara, Hiroko Okada, Eiko Goto, Takahiro Kiuchi

**Affiliations:** Department of Health Communication, School of Public Health, The University of Tokyo, Tokyo 113-8655, Japan; okadahiroko-tky@umin.ac.jp (H.O.); gotoue-tky@umin.ac.jp (E.G.); tak-kiuchi@umin.ac.jp (T.K.)

**Keywords:** HPV vaccination, cervical cancer, health literacy, readability, patient education, written health information, health communication

## Abstract

It is important to provide information on HPV vaccination and on early detection and early treatment for cervical cancer. Readability is a key aspect in the success of cancer communication using written health information. We reviewed studies assessing the readability of information on HPV vaccination and cervical cancer. We conducted a systematic literature search in June 2021 using four online databases (Medline, CINAHL, PsycArticles, and PsycINFO). Studies that assessed the readability level of online and offline information regarding HPV vaccination and cervical cancer were included in the review. Twelve articles were deemed fit for study inclusion. Our results showed that most of the materials assessed were difficult to read and higher than eighth-grade reading level. Few of the materials assessed were at the recommended grade 5–6 level or below. Readability assessments of HPV vaccination and cervical cancer information are scarce. Additional studies on the readability of information regarding HPV vaccination and cervical cancer are recommended. Health professionals should develop health information on HPV vaccination and cervical cancer that is easy to read.

## 1. Introduction

More than 570,000 new cases of cervical cancer are diagnosed and approximately 311,000 women die from the disease annually worldwide [1]. The main cause of cervical cancer is infection with human papillomavirus (HPV). Most cervical cancers can be prevented by vaccination against HPV infection and are curable if detected early in the precancerous stage via screening. It is therefore important to provide people with the information that cervical cancer is a preventable and curable disease and that measures are available for its prevention and early detection. Such HPV vaccination and cervical cancer-related information is often provided as written text in print [2] and as digital media [3]. However, such health information is often written at a level of readability that is difficult for many target audiences to read [4].

Health literacy is “the degree to which individuals have the capacity to obtain, process, and understand basic health information” [5]. A lower level of health literacy is associated with lower vaccination adoption rates and cancer screening adoption rates owing to difficulty with comprehension of the information and complex procedures that are needed to adopt vaccination and screening [6,7]. In the study of health literacy, accessibility and understandability of health information are generally discussed in terms of readability [8]. Health information should be readable to all individuals, regardless of their literacy level. It is recommended that patient educational materials should be written at a fifth- to sixth-grade level or lower [9].

Readability is the reading comprehension level required for a person to understand written materials [10]. Some existing readability assessment tools in English include the Simple Measure of Gobbledygook Grade Level (SMOG), Flesch–Kincaid Grade Level (FKGL) test, Flesch–Kincaid Reading Ease (FRE) test, Fry Readability Graph (FRG), and Gunning Fog Index (GFI) [11,12]. Validated readability assessment tools are also available in other languages such as French [13], German [14], and Spanish [15]. These tools can be used to assess the readability of written text based on factors such as the number of words in a sentence and word difficulty level.

Studies indicate that the readability of information can influence individuals’ understanding and behavior regarding prevention, early detection, and early treatment of cervical cancer. For example, one intervention study showed that participants who were given easy-to-read vaccine information had significantly higher scores of comprehension and recall than those who were given standard materials to read (16.6 vs. 13.9, *p* < 0.001, 15.1 vs. 11.3, *p* < 0.001, respectively) [16]. Another intervention study showed that participants who received materials that were rated easier to read using a readability assessment tool had higher rates of undergoing gynecologic cancer screening than those who received materials that were rated less easy to read (29.4% vs. 14.2%, *p* = 0.007) [17]. Thus, readability is considered an essential quality in the evaluation of HPV vaccination and cervical cancer-related information.

To our knowledge, there are no reviews of readability studies on HPV vaccination and cervical cancer-related information; thus, knowledge on this subject is limited. Therefore, we conducted the present study to provide the first systematic scoping review focused on studies that objectively assessed the readability levels of online and offline information materials related to HPV vaccination and cervical cancer using readability formulas. Our study aims were to create an overview of the published literature and to identify the content and gaps in published studies to help guide future research and practice regarding improvement in HPV vaccination and cervical cancer-related written information. Our research questions were as follows: (1) What is the content and gaps in previous studies assessing the readability of HPV vaccination and cervical cancer information? (2) What was revealed in previous studies regarding the readability levels of HPV vaccination and cervical cancer-related information? (3) What factors affecting the appropriateness of HPV vaccination and cervical cancer information, other than readability level, were evaluated in previous studies, and what were the findings?

## 2. Materials and Methods

### 2.1. Search Strategy

We carried out a systematic literature search on 15 June 2021, following the Preferred Reporting Items for Systematic Reviews and Meta-Analysis (PRISMA) statement [18]. We used the following combination of keywords: (“HPV vaccine” OR “HPV vaccination” OR “human papillomavirus vaccine” OR “human papillomavirus vaccination” OR “cervical cancer”) and “readability.” We searched the resulting titles and abstracts in four online databases (Medline, CINAHL, PsycArticles, and PsycINFO). We performed a search of additional databases (Web of Science, Scopus, Academic Search Complete, and Communication Abstracts) in a more extensive search on 9 September 2021. We did not filter by year, language, or publication type. Additionally, to identify studies that could not be found in the online database search, we manually searched the reference lists of the included studies.

### 2.2. Study Selection

**Figure 1:**
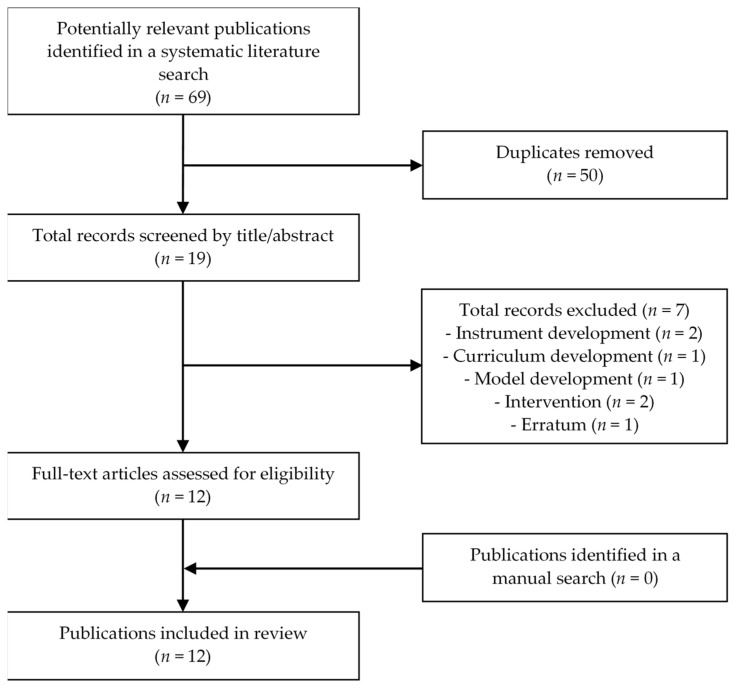
Flow chart of the literature search process.

The inclusion criteria were studies that assessed the readability levels of online and offline informational materials related to HPV vaccination and cervical cancer using readability formulas. The exclusion criteria were studies that did not assess the readability levels of HPV vaccination and cervical cancer-related informational materials, such as those focusing on instrument development and curriculum development (see Figure 1). The first review was done by the first author (TO). Then, the second author (HO) independently reviewed all the articles. Any differences in evaluation between the authors were resolved through discussion.

### 2.3. Data Extraction and Synthesis

For each study, we extracted relevant data, including the year of publication, the country in which the study was conducted, the language in which the study was conducted, the readability formula used, the material evaluated, the provider of the material, and the main results regarding readability level evaluation. When the included studies reported the characteristics of factors affecting the appropriateness of information other than readability level (e.g., content, accuracy), we also extracted those data. The first author (TO) extracted the data using Microsoft Excel (Redmond, WA, USA). The second author (HO) then checked for accuracy and completeness and verified all data extraction. Any differences in data extraction between the authors were resolved through discussion. We aggregated and analyzed the data in line with the study aims. We conducted a descriptive analysis of the included studies, identifying the study characteristics, the results of readability assessment, and other results affecting the appropriateness of information on HPV vaccination and cervical cancer.

## 3. Results

The search yielded 69 articles published between 1992 and 2021. After scrutinizing the relevant titles and abstracts, as well as the full texts, we identified 12 papers that were suitable for inclusion and were subsequently reviewed (see Figure 1).

### 3.1. Study Characteristics

Table 1 provides an overview of the included studies. Seven studies were conducted in the United States [19,20,21,22,23,24,25], three in Canada [26,27,28] one in China [29], and one in Japan [30]. Ten studies conducted in the United States and Canada assessed materials in English; the other two studies assessed materials in Chinese or Japanese language. Regarding content, eight studies assessed HPV vaccination information [19,21,22,23,27,28,29,30]; four assessed cervical cancer information [20,24,25,26]. Regarding format, eight studies assessed online information and websites [19,20,21,23,26,27,29,30]; one assessed printed HPV vaccination counseling materials [22]; one assessed patient consent forms for radiation therapy for cervical cancer [24], one assessed newspaper articles regarding HPV vaccines [28], and one study assessed written materials on cervical cancer prevention [25]. Regarding providers, eight studies reported on informational materials provided by commercial websites [26,29] governments [21,27], gynecologic oncology groups [24], health professionals and non-health professionals [30], national newspapers [28], and multiple sources [25]. The number of materials evaluated in the included studies ranged from 4 to 4928.

### 3.2. Readability Assessment

As Table 1 shows, the 10 studies assessing English materials used the SMOG, FKGL, FRE, GFI, FRG, Dale–Chall formula, Coleman–Liau Index, and the Automated Readability Index to assess readability. Six of ten studies assessing English materials used multiple readability assessment tools. The most frequently used readability formulas were the SMOG and FKGL, which were used in eight and six studies, respectively. Japanese texts were assessed using a readability formula called the jReadability. Readability of Chinese texts was assessed using word levels and character numbers in sentences.

All 10 studies from the United States and Canada reported that most of the information assessed was higher than eighth-grade reading level [19,20,21,22,23,24,25,26,27,28]. These studies showed that there was little information on HPV vaccination and cervical cancer written at the recommended fifth- to sixth-grade level or lower. A study from China reported that 71% of HPV vaccine-related messages were rated at a doctoral readability level and 20% were at undergraduate level [29]. A study from Japan reported that pro-HPV vaccination messages were significantly more difficult to read than anti-HPV vaccination messages [30].

### 3.3. Other Factors

As Table 1 shows, six studies reported the characteristics of informational materials, other than readability levels [22,24,25,26,27,29]. Three studies used the Suitability Assessment of Materials (SAM) [22,24,25]. One of those three studies used the SAM to evaluate patient consent forms to receive radiation therapy for cervical cancer; that study reported that three of the four consent forms scored within the lower portion of the “adequate” range, and one consent form was deemed “not suitable” [24]. One of those three studies evaluated HPV vaccination counseling print materials using the Patient Education Materials Assessment Tool (PEMAT) in addition to the SAM. That study reported that 68% of materials were categorized as “not suitable” using the SAM, and the average PEMAT score was 42%, well below the threshold for high understandability [22]. One of those three studies evaluated comprehensibility of written cervical cancer prevention materials combined with assessment using the SAM. That study reported that 20% of materials were rated “superior,” 68% were “adequate,” and 12% were “not suitable” [25]. One study conducted content evaluation and used the DISCERN score to evaluate HPV vaccine-related online messages: that study reported biases and inaccuracies in content and less than 1% of messages were ranked of good quality [29]. One study evaluated accountability, site interactivity and organization, content coverage, and content accuracy of cervical cancer-related online information. That study reported that much of the evaluated information lacked accountability or recent updates and that important topics, such as prognosis and staging, were underrepresented [26]. One study evaluated text coherence of HPV information websites and reported that HPV vaccine information had a lower level of coherence than that needed for the general public [27].

## 4. Discussion

We systematically reviewed studies assessing readability of HPV vaccination and cervical cancer-related information using readability formulas. We discuss our findings below, in line with our research questions: study characteristics (research question 1); readability level (research question 2); and assessment of factors other than readability level (research question 3).

Regarding study characteristics, readability studies of HPV vaccination and cervical cancer information began relatively recently, in 2009, despite the history and richness of readability studies in the field of public health. Compared with the wealth of readability studies evaluating written health information [31,32], the total of 12 readability studies on HPV vaccination and cervical cancer-related information represents a small number. Studies conducted in the United States and Canada accounted for a large proportion of these studies and assessed English-language text. Many of the included studies assessed online information and HPV vaccine information. No studies were conducted in European countries or in Asian countries other than China and Japan or in low-and middle-income countries. Future studies should assess the readability of HPV vaccination and cervical cancer-related information in languages other than English, Chinese, and Japanese. Future studies should also assess the readability of HPV vaccination and cervical cancer-related information in low-and middle-income countries. Additionally, future studies should assess the readability of information regarding cervical cancer prevention and screen-and-treat approaches; we found only four studies related to this. Four of the 12 included studies did not report the provider of the assessed materials. Readability of health information should be assessed separately by the provider because it is essential to effectively improve the readability of information on HPV vaccination and cervical cancer.

The readability levels of most HPV vaccination and cervical cancer-related information were higher than an eighth-grade level. However, it is recommended that health information should be written at a fifth- to sixth-grade level or lower [9]. Our finding is consistent with those of other studies showing that health information tends to be written at a readability level above high school level [12,33]. Studies that assessed influenza vaccine information reported that the average grade levels of pro-influenza vaccine Facebook posts were higher than an 11th-grade reading level during the study period [34], and pro-influenza vaccine online messages were significantly more difficult to read than anti-influenza vaccine messages [35]. One study reported that all online patient educational materials regarding mammography assessed in that study were written at above a sixth-grade reading level and the average reading levels were higher than 10th grade according to multiple readability formulas [36]. Studies that assessed colorectal cancer screening online information reported that most materials surpassed the sixth-grade reading level [37,38], and the average reading level was 11th grade [38]. Studies that assessed lung cancer screening online information also reported that only a few of the evaluated materials had reading levels at or below a sixth-grade reading level [39,40], and the average level was 10th grade [40]. Thus, information on the HPV vaccination and cervical cancer, as well as information on other types of vaccines and cancer screening, is often written above the recommended sixth-grade reading level and is difficult to read. Vaccine, screening, and treatment information requires a certain level of literacy and numeracy skills to understand, making it particularly difficult to convey information to a general audience with low health literacy [7,41]. Difficulty in reading HPV vaccination and cervical cancer-related information can be a barrier to prevention, early detection, and early treatment of cervical cancer [16,17]. Written information on HPV vaccination and cervical cancer must be conveyed in an easy-to-read manner in terms of health literacy.

The ease of reading information about HPV vaccination and cervical cancer is also important in terms of processing fluency. Processing fluency refers to the ease with which information can be processed [42]. Studies of processing fluency indicate that easy-to-read text is better liked and more trusted than difficult-to-read text [42]. Judgment is affected not only by the content of thoughts but also by metacognitive experiences in processing those thoughts [43]. Processing fluency is a metacognitive cue that influences judgment [43]. One review of studies investigating processing fluency argues that fluently processed material leads to a more favorable attitude among readers [44]. For example, one study reported that participants had more trust in easy-to-read material than difficult-to-read material [45]. If HPV vaccination and cervical cancer-related information is easy to read, it may increase trust in the information among readers. Thus, toward successful communication, the readability of HPV vaccination and cervical cancer information is important to generate desirable attitudes toward prevention, early detection, and early treatment of cervical cancer.

Regarding the evaluation of factors other than readability level, we found that six of the twelve included studies evaluated readability levels only. However, multiple factors affect the ease of reading and understanding information on HPV vaccination and cervical cancer, in addition to readability levels assessed using readability formulas. Three studies used the SAM [22,24,25]. The SAM evaluates written health materials in domains such as content, literacy demand, graphics, layout and typography, and learning simulation and motivation [46]. A SAM score of 70–100 is considered “superior,” 40–69 is deemed “adequate,” and scores <40 are considered “not suitable.” [46] One of those three studies used the PEMAT in addition to the SAM [22]. The PEMAT assesses understandability of health materials in terms of content, word choice and style, use of numbers, organization, layout and design, and use of visual aids [47]. A score of greater than 70% indicates greater understandability [47]. These three studies indicated that a certain proportion of HPV vaccine information is inappropriate in terms of suitability and understandability; these studies reported that much of the evaluated HPV vaccination and cervical cancer-related information used technical terms, did not use visual aids, and lacked motivators to adopt health behaviors and persuasive techniques [22,24,25]. These characteristics would make the information on HPV vaccination and cervical cancer difficult to read and understand by lay audiences with inadequate health literacy. Additionally, these characteristics would make it difficult for lay audiences to adopt optimal behaviors.

A study in China evaluated the content and DISCERN score as well as readability of HPV vaccine-related online messages [29]. Content was evaluated using a checklist of information topics under four major themes: (1) basic information, (2) the HPV vaccine and its functions, (3) inoculation and precautions related to vaccination, and (4) quality, safety, and side effects. In that study, most messages explained the HPV vaccine and its function, yet fewer than half explained its safety and side effects. The content accuracy was not optimal, with 45% of the included messages having errors. Additionally, that study evaluated DISCERN scores. The DISCERN scale includes three parts: publication reliability as a source of information, quality assessment of information, and overall quality ratings of the publication [48]. The total score of all messages is calculated and classified as poor (<40%), fair (40–79%), and good (>79%) [48]. In one study, 17% of messages were poor quality, 83% were fair quality, and only one message (<1%) was good quality [29]. A study in Canada evaluated cervical cancer-related online information in terms of accountability, site interactivity and organization, content coverage, and content accuracy as well as reading level [26]. Those items were evaluated using a tool developed by the authors referring to the Health on the Net Foundation Code of Conduct (HONcode) principles and the DISCERN scale. Although most websites were accurate in terms of information, many lacked accountability or recent updates. The usability and interactivity of websites was high. Although content such as cervical cancer screening and prevention was often covered, content such as prognosis and stage of disease, which are considered important for patients with cervical cancer, was infrequently covered. Thus, these two studies reported issues regarding accountability, content, and accuracy of HPV vaccination and cervical cancer-related information.

As these studies show, using broader assessment tools such as the SAM [46], the PEMAT [47], and the DISCERN scale [48] enables objective evaluation of the quality of both HPV vaccination and cervical cancer information, in addition to the assessment of readability levels. Such extensive evaluation of information related to HPV vaccination and cervical cancer will contribute to improving the health information provided to patients and the general public so as to promote prevention, early detection, and early treatment of cervical cancer.

Written information is widely used in cancer communication provided by health professionals to their patients and to the general public regarding prevention, early detection, and early treatment. Readability is one of the keys to successful communication regarding cervical cancer using written information. We call for further studies on improving the readability of HPV vaccination and cervical cancer-related information and suggest the following questions, which should be addressed in future research. What is the readability level of HPV vaccination and cervical cancer-related information materials that have not been assessed in previous studies? What is the readability level of non-English, non-Chinese, and non-Japanese HPV vaccination and cervical cancer-related information? What is the readability level of HPV vaccination and cervical cancer-related information materials in low-and middle-income countries? What factors are present apart from readability level, such as ease of understanding and appropriateness of HPV vaccination and cervical cancer-related information? What is the gap between the reading level of the target audiences and the level needed to read HPV vaccination and cervical cancer-related information? Future studies should answer these research questions.

Regarding practices to make information on HPV vaccination and cervical cancer easier to read, guidelines are available such as the SAM [46], the PEMAT [47], the Centers for Disease Control and Prevention (CDC) Clear Communication Index [49], and the CDC Simply Put guide [11]. These guidelines recommend modifying technical terms to everyday language, shortening long sentences, arranging information in a logical order, adding informative headers to each section, and including a clear summary. Health professionals should refer to such recommendations so as to develop HPV vaccination and cervical cancer-related information that is easy to read and understand. Health professionals can also use readability assessment tools to make HPV vaccination and cervical cancer-related text more readable. Validated readability assessment tools such as the SMOG and FKGL are available on the Internet [11,12]. When HPV vaccination and cervical cancer-related information is rated difficult to read, the text should be revised to use plain language, active voice, and short words and sentences [11,12]. Health professionals should create readable HPV vaccination and cervical cancer information using such guidelines and tools to help audiences adopt optimal judgment and behaviors with respect to prevention, early detection, and early treatment of cervical cancer.

There are several limitations in this study. Although we systematically searched for related studies in multiple databases using a combination of appropriate keywords, we may have missed some publications, such as studies that assessed readability as part of a larger analysis. In this study, we focused on the level of readability as calculated using a readability formula. However, there are other factors affecting the readability and understandability of HPV vaccination and cervical cancer-related information. Because the readability formulas used were different for English, Chinese, and Japanese, the way in which the readability level was judged differed depending on the language. Despite these limitations, this was the first systematic scoping review of readability assessment studies on HPV vaccination and cervical cancer-related information. The present study findings have implications for further research and practice aiming to improve written health information on HPV vaccination and cervical cancer.

## 5. Conclusions

This systematic literature review showed that readability assessment of HPV vaccination and cervical cancer-related information is scarce. This review also found that the readability level of most HPV vaccination and cervical cancer-related information is higher than what is recommended. In addition to readability levels as assessed using various formulas, issues related to multiple factors that affect readability, understandability, and suitability of HPV vaccination and cervical cancer-related information are proposed. More extensive research is needed to understand the current status regarding readability of HPV vaccination and cervical cancer-related information. Improving the readability of HPV vaccination and cervical cancer-related information will help patients and the general public to make the best decisions and adopt optimal behaviors regarding prevention, early detection, and early treatment of cervical cancer.

## Figures and Tables

**Table 1:** Studies assessing readability level of HPV vaccination and cervical cancer-related information.

Reference	Year	Country	Language	Readability Formula	Material (n)	Provider of Material	Main Results	Assessment of Factors Other than Readability
Wang et al. [29]	2021	China	Chinese	Calculated using word levels and character numbers in sentences	HPV vaccine-related online messages (294)	Most (92%) messages were from commercial websites.	The readability level of 71% of messages were rated at doctoral level, and 20% were undergraduate level.	There were biases in the content of the message. Only 55% of messages had no errors. Regarding the DISCERN scores, only one message (<1%) had good quality.
Dawson et al. [26]	2020	Canada	English	SMOG, FKGL,	Cervical cancer-related online information (100)	42% of websites were commercial, followed by those of non-profit organizations, government, and academic centers.	More than 95% of websites were at a high school reading level (8th grade) or higher.	Many lacked accountability or recent updates. Usability and interactivity were high. Important topics such as prognosis and staging were underrepresented.
MacLean et al. [19]	2019	United States	English	SMOG, FKGL, FRE, GFI, CLI,	HPV vaccination websites (100)	Not reported	75% of websites rated difficult to read (>10th grade). Only a few websites were rated easy to read (<6th grade).	None
Martin et al. [20]	2019	United States	English	SMOG, FKGL, GFI, DC	Cervical cancer patient education online materials (4928)	Not reported	Mean grade-level readability was 8.9, i.e., a high school reading level.	None
Tulsieram et al. [27]	2018	Canada	English	SMOG, GFI	Provincial department/Ministry of Health HPV information websites (7)	Provincial governments	Most (six of seven provinces) websites were rated as difficult to read (>12th grade).	Text coherence was not adequate for lay individuals to understand.
Calo et al. [21]	2018	United States	English	SMOG, FKGL, GFI, CLI, ARI	HPV vaccination messages online (267)	Government, medical association, Medscape, medical journals, educational clearinghouses	The readability level of most materials (62%) was ≥9th grade. Only 12% were easy to read (≤6th grade).	None
Chhabra et al. [22]	2018	United States	English	SMOG, FKGL, FRE, GFI, FRG	HPV vaccination counseling print materials (38)	State government	Four documents (10.5%) were at a 6th-grade reading level or lower, and 15 documents (39.5%) at a 10th-grade or higher reading level.	68% of materials were categorized as “unsuitable” with the SAM. Mean PEMAT score was 42%, which was much lower than the threshold for high understandability.
Okuhara et al. [30]	2017	Japan	Japanese	jReadability	Pro-and anti-HPV vaccination online messages (270)	Health professionals and non-health professionals	Pro-vaccination messages were difficult to read. Anti-vaccination messages were significantly easier to read than pro-vaccination messages.	None
Fu et al. [23]	2016	United States	English	FKGL	Critical and noncritical HPV vaccination web pages (116)	Not reported	Most web pages required a 12th-grade reading level.	None
MacDougall et al. [24]	2012	United States	English	SMOG	Patient consent forms for radiation therapy for cervical cancer (4)	Gynecologic oncology group	Readability ranged from grades 12.18 to 16.13; required at least a high school education.	Three of four consent forms scored in the lower portion of the “adequate” range, and one consent Form was “not suitable” using the SAM.
Abdelmutti and Hoffman-Goetz [28]	2009	Canada	English	SMOG	Newspaper articles on HPV vaccines (164)	National newspapers	The readability of the article rated as inadequate (>8th-grade level).	None
Helitzer et al. [25]	2009	United States	English	FRG	Cervical cancer prevention written materials (69)	Web-based fact sheets, magazine articles, advertisements, health system forms, books, newspaper articles	Most materials were written at too high a readability level. The mean reading level was 11th grade.	20% of materials were rated “superior,” 68% were “adequate,” and 12% were “not suitable” using the SAM and comprehensibility assessment.

Abbeviations: HPV, human papillomavirus; SMOG, Simple Measure of Gobbledygook Grade Level; FKGL, Flesch–Kincaid Grade Level; FRE, Flesch–Kincaid Reading Ease; FRG, Fry Readability Graph; GFI, Gunning Fog Index; DC, Dale–Chall formula; CLI, Coleman–Liau Index; ARI, Automated Readability Index; SAM, Suitability Assessment of Materials; PEMAT, Patient Education Materials Assessment Tool.
